# Suicide on the Railways in Belgium: A Typology of Locations and Potential for Prevention

**DOI:** 10.3390/ijerph15102074

**Published:** 2018-09-21

**Authors:** Mathieu Strale, Karolina Krysinska, Gaëtan Van Overmeiren, Karl Andriessen

**Affiliations:** 1 Institut de Gestion de l’Environnement et d’Amenagement du Territoire (IGEAT-ULB), Université Libre de Bruxelles, 1050 Brussels, Belgium; mstrale@ulb.ac.be; 2 Centre for Primary Health Care and Equity, University of New South Wales, Sydney NSW 2052, Australia; k.krysinska@unsw.edu.au; 3 Faculty of Psychology and Educational Sciences, KU Leuven-University of Leuven, 3000 Leuven, Belgium; 4 Centre for Mental Health, School of Population and Global Health, The University of Melbourne, Parkville VIC 3010, Australia; 5 Infrabel, Operational Safety Division, 1060 Brussels, Belgium; gaetan.vanovermeiren@infrabel.be; 6 School of Psychiatry, University of New South Wales, Sydney, Hospital Rd, Randwick NSW 2031, Australia

**Keywords:** Belgium, geographical distribution, principal component analysis, psychiatric hospitals, railway, suicide, prevention

## Abstract

Suicide on railway networks comprises a serious public health problem. However, the geographical distribution and the environmental risk factors remain unclear. This study analyzed the geographic distribution of railway suicides in Belgium from 2008–2013 at the level of a railway section (average length of 3.5 km). Principal component analysis (PCA) identified three groups of correlations that helped explain the variance of railway suicide. The three groups are related to characteristics of urban spaces, psychiatric facilities, and railway traffic density. Based on the PCA results, the study found four types of railway sections. The density of railway suicide was average and low in the urban and rural/industrial sections, respectively. However, it was high in the suburban sections and the sections close to psychiatric facilities. As the geographical proximity of a psychiatric facility comprises a specific risk factor for suicide on railways, preventative measures should target these sections and establish collaborations with psychiatric facilities. The typology of locations found in this study constitutes crucial information for national and local suicide prevention on the Belgian railway network.

## 1. Introduction

Suicide on railway networks is a major public health problem and accounts for 1.3% (Canada) to 12.3% (The Netherlands) of all suicides across countries worldwide [1]. In addition to comprising a serious safety problem, suicides on railways are likely to occur in public spaces, potentially affecting many people. Suicides on railways are associated with mental health ramifications (e.g., psychological trauma) to train drivers, passengers, witnesses, first responders, and bereaved relatives; economic costs due to delays and medical and police interventions; and intangible costs related to the loss of life [2,3]. Typical preventative measures include restricting access to the tracks (both on open tracks and in stations), the installation of security cameras or blue lights, suicide prevention training of railway staff, collaboration with primary stakeholders (police, hospitals in the vicinity of the rails), public safety messages, and tuning down media coverage of railway suicides [4,5]. The strongest evidence of effectiveness has been reported for fencing and other physical barriers and responsible media reporting, though few studies have examined the effects of multiple measures [4]. It has also been argued that the research on the prevalence of rail suicide should include an assessment of the local environment to tailor the prevention measures [1]. 

Approximately 5.3% of all suicides in Belgium (total suicide rate 17 per 100,000 inhabitants in 2014; most recent data [6]) occur on railways [7]. An earlier study by our group found differences in the geographical distribution of rail suicide in Belgium on the arrondissement-level [8]. The density of railway suicide was higher in the more densely populated northern part of Belgium, Brussels and major towns in Flanders (northern part of Belgium), and in Wallonia (southern part of Belgium). Although rail suicides were frequent in urban areas, the ratio of rail suicides per inhabitant was higher in suburban areas, and the number and the ratio of rail suicides were low in rural areas. In addition, up to one third of railway suicides in the country appear to cluster in specific areas [7]. 

Given the potential for suicide prevention in these areas, we assessed the environment of all locations (*N* = 43) where at least two suicides occurred in a 2-km railway section over a five-year period (2003–2009) [9]. All these high-risk locations were easily accessible as they offered privacy and an opportunity to hide for a person contemplating suicide, and provided only limited visibility to a train driver. Additionally, many high-risk locations (39%) were less than 2 km from a mental health facility [9]. While this initial study provided useful information regarding the characteristics of high-risk locations in Belgium, it was limited to field observations and information gathered from local stakeholders and did not include a systematic analysis of environmental factors, such as population or railway density [9]. Still, our findings were in line with other studies, which have found a link between the presence of psychiatric facilities, easy access to tracks, and the distribution of railway suicides [1,10,11,12]. 

The current study aims to further analyze the geography of suicide on the Belgian railway network and to propose a typology of railway suicide locations in the country using principal component analysis (PCA) [8]. PCA has already been used successfully to study the factors that explain the geography of suicide [8,11] and is an effective method for analyzing social phenomena and identifying their explanatory factors [13,14]. The variables of interest in this study include the intensity of railway traffic, the density of access to railways (i.e., bridges, stations, and railroad crossings), hospital and school infrastructure, population density, and number of psychiatric beds available. These variables have been found to relate to suicide on the railway networks in Belgium [8], and internationally [1,5]. Infrabel, the Belgian railway manager, has noted a greater number of trespassing near schools; however, it is not known if this constitutes a risk factor for railway suicide [15].

## 2. Materials and Methods 

### 2.1. Study Area and Level of Analysis 

The study area was Belgium, a federal state with a total population of 11.2 million inhabitants (1 January 2014) and covering a territory of 30,528 km^2^. Both the Flemish (northern part of the country) and the Walloon (southern) regions consist of five provinces each: Antwerp, East-Flanders, Flemish-Brabant, Limburg, and West-Flanders; and Hainaut, Liège, Luxembourg, Namur, and Walloon-Brabant, respectively. The density of the rail network reflects the repartition of the Belgian population and employment [7]. The rail network is denser in the northern part and in the center of the country, around the capital region of Brussels. The rail infrastructure in Wallonia is less developed; the main rail link there is the West-East urban continuum, linking Mons, Charleroi, Namur, and Liège. 

Analyses were conducted at the level of a railway section, i.e., tracks between two characteristic points (PTCARs). A PTCAR represents an imaginary line across tracks in a station, a siding, or a border point, which acts as a reference point for the planning and real-time monitoring of a train. The Belgian railway network comprises 1285 PTCARs with an average length of 3.5 km. A buffer of five km was applied around each railway section, which was considered as the area of influence around railways and railway stations [16]. We calculated the value of explanatory variables in these buffers.

### 2.2. Materials

The values of explanatory variables were calculated either at the level of a railway section or a buffer around a railway section. The following variables were included in the analysis: (a) density of railway suicide; (b) railway traffic (i.e., the number of trains per day and per section); (c) density of access to the railway network (bridges, stations, railroad crossings); (d) density of population; (e) density of schools; (f) density of hospitals; and (g) density of psychiatric beds (Table 1). Infrabel, the Belgian manager of the rail infrastructure, provided data on suicide on railways from 2008–2013, rail infrastructure, and traffic. The Belgian Federal Ministry of Health provided hospital-related data. Data on schools were obtained from Walloon and Flemish governmental websites [17,18]. Data on population density per square kilometer in 2012 (most recent data) was provided by StatBel [19]. Based on these data, we calculated population density in the buffers around railway sections. 

### 2.3. Analyses 

Principal component analysis (PCA) was used to analyze the data, and to identify the geographic distribution of railway suicides and the explanatory factors at the railway section level. The PCA identifies coherent information contained in the initial variables. The PCA works as if each initial variable was a dimension of the initial cloud of information, i.e., the departing matrix. Firstly, based on correlation factors between initial variables and the variance of the observations of these variables, the PCA identifies the statistical links between the initial variables, highlights the main coherent information, and detects strong patterns contained in the initial variables. 

**Figure 1:**
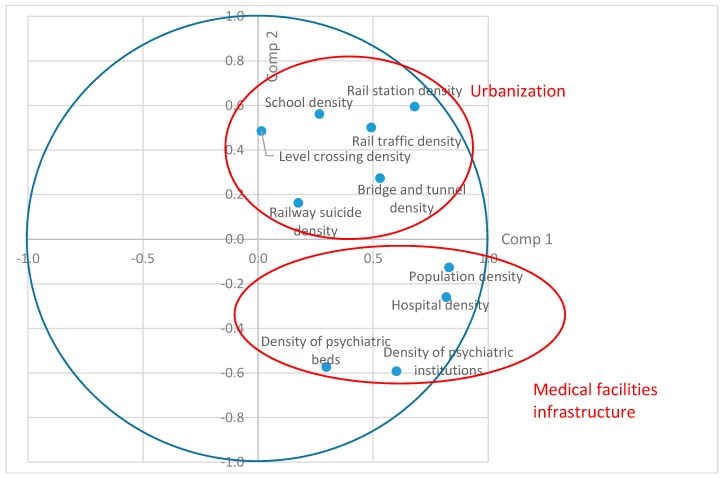
Saturation levels on Component One and Component Two.

Secondly, the PCA builds new synthetic variables, i.e., components, in order to maximize the coherence of information they contain (which is based on the variance). The components are independent from each other. There is a hierarchical classification: the first component gathers more information than the second, the second more than the third, etc. Correlation coefficients are calculated between the initial variables and the new components (i.e., saturation levels). The saturation levels of initial variables on the components are useful for understanding the meaning of these new synthetic variables. The saturation levels can be analyzed as if they were correlation factors between the initial variables and the new components, and their values range between −1 and 1. A positive saturation value on one component means that the variable is positively correlated with the component; a negative value indicates a negative correlation. The projection of saturation levels of Components One and Two (Figure 1) allows identifying groups of initial variables which similarly correlate with the components.

Finally, based on the values of the initial variable, the initial observations were positioned on new components (i.e., scores of observations on components). Calculations of these scores were based on a multivariate correlation between the initial situation of observations regarding the departing variables and their position on the new components. Observations with positive scores have a value above the average on variables that are also positively correlated with the component and have a value below the average on variables that are negatively correlated with the component. Based on the PCA results, the study built a typology using the Ward Hierarchical method. The aim was to identify coherent territorial patterns of railway suicides in Belgium, which are based on initial variables. The analysis was conducted using R statistical freeware [20] and the maps were built using QGIS Geographic Information System freeware [21].

## 3. Results

**Figure 2:**
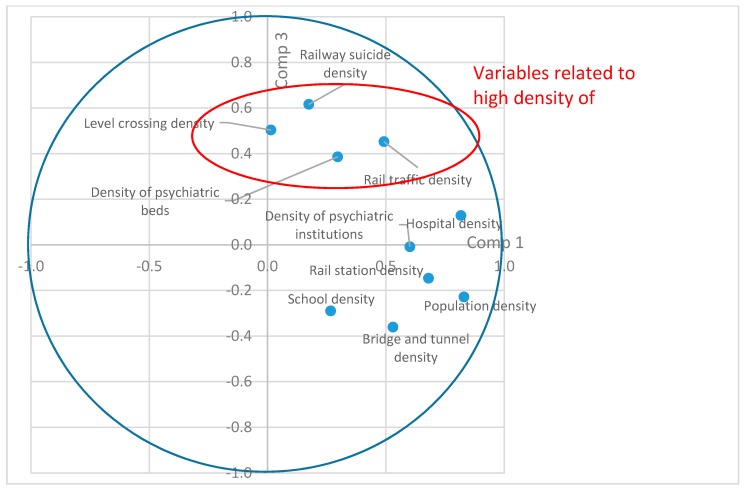
Saturation levels on Component Three.

Components One, Two, and Three were analyzed, which accounted for 63% of the total variance, i.e., information contained in the initial matrix. Additional components accounting for less variance were excluded. Based on the results of Component One and Component Two, two groups of variables appeared (Figure 1). The first group of variables related to urbanization and encompassed the density of rail traffic, rail stations, level crossings, and bridges and tunnels, as well as population density and density of railway suicide. Belgium has very few railways sections in mountains; hence, bridges and tunnels are concentrated in urban areas. The second group was related to medical infrastructure, and encompassed the density of hospitals, psychiatric beds, and psychiatric facilities. Population density was an indicator of both urbanization and the density of medical infrastructure. Component Three was positively correlated with variables related to a high density of railway suicides, railway traffic, and level crossing density (Figure 2). School density was not included among the variables related to a higher suicide density.

### Toward a Typology of Suicide on the Railway Network in Belgium

**Figure 3:**
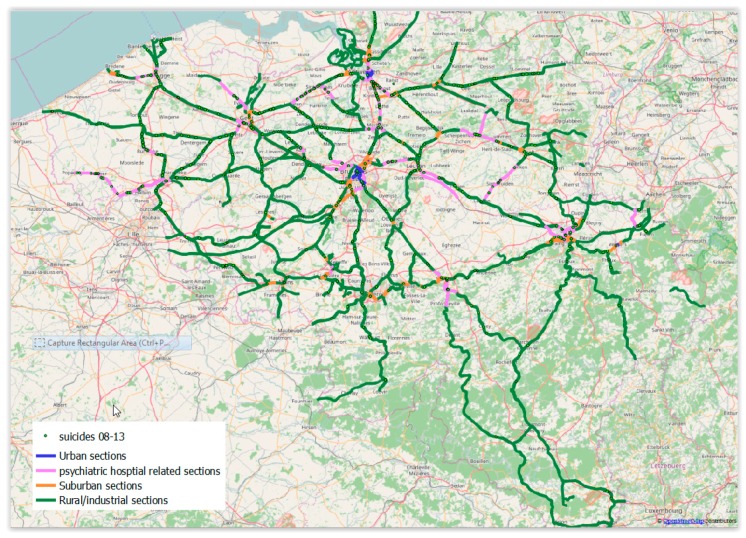
Four types of railway sections related to suicide on railways in Belgium.

Using the Ward Hierarchical method and based on the scores of Components One, Two, and Three emerging from the PCA, a typology of suicide on the Belgian railway network can be proposed (Figure 3, Table 2). This typology includes four types of railway sections: (a) urban sections with dense railway traffic, dense population and railways infrastructure, and average density of railway suicide; (b) suburban sections with dense railway traffic, high density of accesses to the railway network, higher average train speed, and high density of railway suicides; (c) railway sections close to psychiatric facilities with a high density of hospitals and psychiatric beds and a high density of railway suicides; (d) rural and industrial railway sections with a low density of railway traffic, a low density of population, and a low density of railway suicides.

## 4. Discussion

This study on suicide on the railway network in Belgium found three groups of variables. These were related to the level of urbanization, the level of infrastructure of medical facilities, and railway variables. There seem to be two groups of explanatory variables of railway suicides. There is an urban group with frequent railway suicides because of the density of population, railway network, and traffic, and due to the easy access to the railway network. Another group is related to psychiatric and medical facilities. Even if the population and the railway traffic and infrastructure are less dense, railway suicides remain numerous. In other words, there is a statistical link between the presence of psychiatric facilities, which entails the presence of individuals at risk of suicide, and the density of railway suicides.

In addition, based on the existing data and using the Wald Hierarchical method, railway sections in Belgium can be divided into four categories in relation to the density of railway suicide. The proposed typology allows the identification of coherent territorial patterns of railway suicides in the country. Suburban sections and railway sections close to psychiatric facilities have a high density of railway suicides, while urban sections have an average density of railway suicides. In contrast, rural and industrial railway sections have a low density of railway suicides. In other words, this study found a high concentration of railway suicides in suburban spaces, and less suicide in dense urban areas and rural spaces. Some factors may help to explain these findings. In suburban areas, rail traffic is dense, trains run at higher speed than in cities, and there is relatively easy access to the network because of level crossings and bridges or tunnels. In dense urban areas, social control is stronger and trains run at lower speeds, reducing the possibility of suicides even if there is easy access to the railways. Finally, rural and industrial areas have a sparsely populated rail network and low traffic, which also significantly reduces the risk of railway suicide [8]. International studies also report on links between population and railway traffic density and railway suicide [1,22,23], indicating ease of access to lethal means as an important risk factor.

A crucial finding of this study is the geographical link between suicides on the railways and psychiatric centers. The proximity of railway tracks to a psychiatric facility is also an important risk factor internationally [1,10,11,12,22,23]. The findings of this research have inspired Infrabel to implement a series of preventative measures focused on rail tracks, railway stations, and the wider railway environment. Since 2012, Infrabel has built an action plan against suicide on the railway network [24]. This action plan is based on a multi-step approach designed in the Campaigns and Awareness Raising Strategies in Traffic Safety project [25] and comprises six steps and their subsequent actions. This approach was also used in the 2011–2014 Reduction of Suicides and Trespasses on Railway property (RESTRAIL) project led by the International Union of Railways [15]. Implemented preventive measures include the installation of fences in open railway lines, establishing collaboration with the psychiatric facilities nearby the railways, installation of blue lights in hotspots stations, installation of intelligent thermal cameras, and poster campaigns at railway stations [24]. All these measures will be evaluated over the next years. 

Although the study findings are important, these must be interpreted with a few limitations. The study was focused on railway suicide in one country (i.e., Belgium). Hence, it is unknown if study findings apply to other types of traffic, such as roads or car traffic, or to other countries.

## 5. Conclusions

This study found four types of locations of railway suicides based on variables related to urbanization, medical and psychiatric facilities, and railway characteristics. Railway sections close to psychiatric facilities and in suburban areas had high density of suicides on the railways, contrary to urban, rural, and industrial railway sections. Given the high suicide risks of these railway sections and the devastating effects on train personnel, passengers, and bereaved family members, comprehensive railway suicide prevention should target these sections and establish collaborations with psychiatric facilities nearby railway tracks. 

## Figures and Tables

**Table 1:** Study variables.

Variable	Scale	Mean Value per Railway Section or Buffer	Standard Deviation
Density of railway suicide	Railway section	0.5 suicides	0.42
Railway traffic (per day)	Railway section	23 trains per day	41.69
Density of access to railway network: level crossings	Railway section	0.01 level crossings	0.01
Density of access to railway network: bridges and tunnels	Railway section	0.01 bridges and tunnels	0.02
Density of access to railway network: railway stations	Railway section	0.01 stations	0.01
Density of population	Buffer around railway section	450 inhabitants	410
Density of schools	Buffer around railway section	0.5 schools	0.19
Density of hospitals	Buffer around railway section	0.12 hospitals	0.78
Density of psychiatric facilities	Buffer around railway section	0.03 psychiatric facilities	0.07
Density of psychiatric beds	Buffer around railway section	0.1 beds	0.13

Sources: Manager of the Rail Infrastructure (Infrabel), Belgian Federal Ministry of Health, http://www.federation-wallonie-bruxelles.be/ [17], https://www.vlaanderen.be/nl [18], StatBel [19].

**Table 2:** Representation of explanatory variables in the proposed typology.

	Rail Traffic Density	School Density	Hospital Density	Railroad Crossing Density	Density of Access to the Rail Network	Density of Bridges and Tunnels	Rail Station Density	Density of Psychiatric Facilities	Population Density	Density of Psychiatric Beds	Density of Railway Suicides
Urban section	2.405	3.276	4.143	0.502	2.805	3.333	2.740	4.440	3.640	2.645	0.878
Suburban section	1.918	0.895	0.607	2.202	1.320	1.198	1.311	0.000	0.882	0.274	1.639
Psychiatric hospital-related section	0.753	1.343	1.956	0.496	0.401	0.455	0.635	3.293	1.101	3.974	1.525
Rural/industrial section	0.314	0.376	0.236	0.926	0.311	0.305	0.384	0.040	0.353	0.082	0.542

Yellow: statistically significant overrepresentation; blue: statistically significant underrepresentation; (significance threshold = 5%).
